# Patterns of yeast diversity distribution and its drivers in rhizosphere soil of Hami melon orchards in different regions of Xinjiang

**DOI:** 10.1186/s12866-021-02222-1

**Published:** 2021-06-06

**Authors:** ShanShan Zhu, YongHui Lei, Chong Wang, YuMei Wei, ChunCheng Wang, YanFei Sun

**Affiliations:** 1 College of life Science / Xinjiang Production and Construction Corps Key Laboratory of Oasis Town and Mountain-basin System Ecology, North 4 Street, College of life Science, Shihezi University, Shihezi, 832003 People’s Republic of China; 2 Department of Plant protection, College of Agriculture, Shihezi University, Shihezi, Xinjiang, 832000 China; 3 Urumqi Customs technique center, Urumqi, 830063 China

**Keywords:** Yeast diversity, Rhizosphere soil, Hami melon orchard, High-throughput sequencing, Environmental factors

## Abstract

**Background:**

The unique climatic conditions of the Xinjiang region nurture rich melon and fruit resources, the melon and fruit sugar sources provide sufficient nutrients for the survival of yeast, and the diverse habitats accompanied by extreme climatic conditions promote the production of yeast diversity and strain resources. However, the relationship between yeast species and their relationship with environmental factors in the soil of Xinjiang specialty cash crop Hami melon is not clear. Here, we aimed to characterize the diversity, community structure, and relationship between yeast species and environmental factors in Hami melon orchards soils in different regions of Xinjiang, China.

**Results:**

Based on Illumina MiSeq high-throughput sequencing analysis of the D1 domain of the LSU rRNA genes, the community richness of yeast in the soil of Northern Xinjiang was higher than in the Southern and Eastern Xinjiang, but the community diversity was significantly lower in the Northern Xinjiang than in the Southern and Eastern Xinjiang. A total of 86 OTUs were classified into 59 genera and 86 species. Most OTUs (90.4%) belonged to the Basidiomycota; only a few (9.6%) belonged to Ascomycota. The most dominant species in the Southern, Eastern and Northern Xinjiang were *Filobasidium magnum* (17.90%)*, Solicoccozyma aeria* (35.83%) and *Filobasidium magnum* (75.36%), respectively. Principal coordinates analysis (PCoA) showed that the yeast community composition in the soils of the three regions were obviously different, with the Southern and Eastern Xinjiang having more similar yeast community. Redundancy analysis (RDA) showed that soil factors such as conductivity (CO), total phosphorus (TP) and Total potassium (TK) and climate factors such as average annual precipitation (PRCP), relative humidity (RH) and net solar radiation intensity (SWGNT) were significantly correlated with yeast communities (*P* < 0.05).

**Conclusion:**

There are abundant yeast resources in the rhizosphere soil of Hami melon orchard in Xinjiang, and there are obvious differences in the diversity and community structure of yeast in the three regions of Xinjiang. Differences in climatic factors related to precipitation, humidity and solar radiation intensity and soil factors related to conductivity, total phosphorus and total potassium are key factors driving yeast diversity and community structure.

**Supplementary Information:**

The online version contains supplementary material available at 10.1186/s12866-021-02222-1.

## Background

Yeast, a common taxon found in the soil, plays an important role in maintaining the ecological functioning of the soil, promoting plant growth, and protecting plants from pests and diseases [1]. Yeasts isolated from soil (e.g., *Filobasidium magnum*, *Naganishia albida*, and *Lipomyces* spp.) have been found to produce extracellular polymeric substances to resist extreme external environmental disturbances, forming soil aggregates in the process and enhancing the stability of the soil structure [2–4]. Plant roots support the survival of yeast species by secreting carbohydrates and organic acids (i.e., amino acids and carboxylic acids). Yeast, in turn, contributes to plant growth and development by dissolving large amounts of nutrients, such as phosphorus and calcium [5–8]. Additionally, some soil yeasts are also present as antagonists of pathogens, such as *Verticillium dahliae* and *Pythium aphanidermatum*, and thus protect the plant from diseases [8, 9]. The size, diversity, and structure of the soil yeast community are known to be influenced by factors, such as soil type, plant species, and geographic location [1]. Moreover, special ecological environments can help yeast species develop tolerance to conditions, such as high / low temperatures-tolerant, drought-tolerant, salinity, etc. [10]. For example, psychrophilic yeasts can be isolated from glaciers [11]. Therefore, the study of yeast diversity, community structure and adaptation strategies in soils under special environments is essential for the development and utilization of yeast resources.

Xinjiang is located in the hinterland of Eurasia, a transition zone between the dry summer zone of Europe and the humid summer belt of East Asia [12]. The special climatic conditions of this region, such as large differences in temperature between day and night and its long hours of daylight, promote the richness of melon and fruit resources [13]. Rich sugar sources in orchard ecosystems promote yeast survival. Meanwhile, the harsh natural environment of dry summers and cold winters has contributed to the evolution of yeast and thus to the accumulation of yeast diversity [12]. Hami melons are popular worldwide and are considered to be a national geographic product and the king of melons in China due to their pleasant aroma, crisp taste, sweetness, and color [14]. The central production areas of Hami melon are the Turpan-Hami Basin, the northwestern and southwestern Tarim Basin, and the north slope of Tianshan Mountain [15, 16]. Currently, the research on Hami melon yeast is mainly focused on the screening of antagonistic yeast to prevent postharvest diseases and control the bacterial fruit blotch disease [17–21]. However, the diversity and composition of yeasts and the ecological factors that influence the yeast community in the soil of Hami melon orchards in different areas of Xinjiang are unknown; such information will provide an in-depth understanding of the adaptation mechanism of Hami melon soil yeast species and the collection and collation of yeast resources in Xinjiang.

**Fig. 1:**
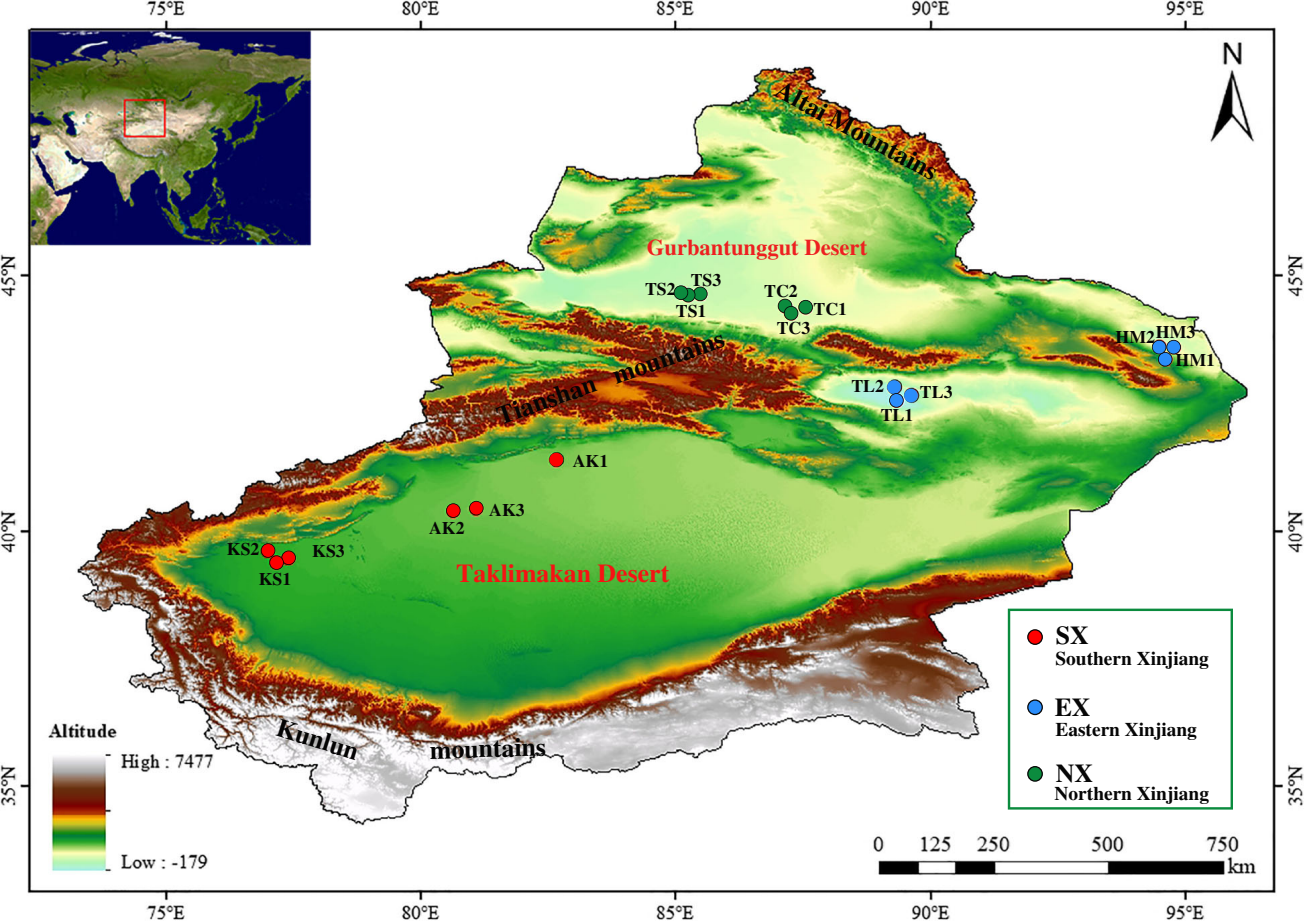
Sampling locations and geographic distribution of all rhizosphere soil samples of Hami melon in Xinjiang, China. Here, SX, EX, and NX represent the sampled areas of Hami melon in Southern Xinjiang, Eastern Xinjiang, and Northern Xinjiang respectively; KS, AK, TL, HM, TC and TS represents the sampled locations in Kashgar and Aksu Prefecture of Southern Xinjiang, Turpan and Hami Prefecture of Eastern Xinjiang, Changji and Shihezi Prefecture of Northern Xinjiang, respectively. Each sample had three replicates (Not shown in the figure). Digital Elevation Model (DEM) provided by the Chinese Academy of Sciences Geospatial Data Cloud Platform

In recent years, research on the yeast species from orchard soils has been done using the culture-dependent method. This method is useful for isolating diverse yeast cultures, enriching the resources bank of yeast strains, screening of useful strains for food, industry, medicine, etc.; however, only a few yeast species have been identified in soil samples using culture-dependent methods, and the possibility for studying microbial population dynamics in an individual environment is limited compared with culture-independent methods [22, 23]. Illumina MiSeq high-throughput sequencing is a technology that is now more widely used, which allows comprehensive and accurate detection of the species composition, generates large data volume with greater coverage compared to traditional culture methods [24]. However, its long run times and short read lengths are not optimal for small-scale sequencing [25, 26]. This study aimed to quantitatively analyze the diversity and structure of rhizosphere soil yeast communities in Hami melon orchards in different regions of Xinjiang (Fig. 1) using the Illumina MiSeq high-throughput sequencer and to explore the environmental factors that influenced the differences in the formation of yeast community structures in different regions. Our results offer new insights into the diversity and structure of yeast communities in the soil of Hami melon orchards in different regions of Xinjiang, providing supplemental information on the yeast resources in Xinjiang orchards.

## Results

### Sequencing analysis and the richness of yeast communities

**Fig. 2:**
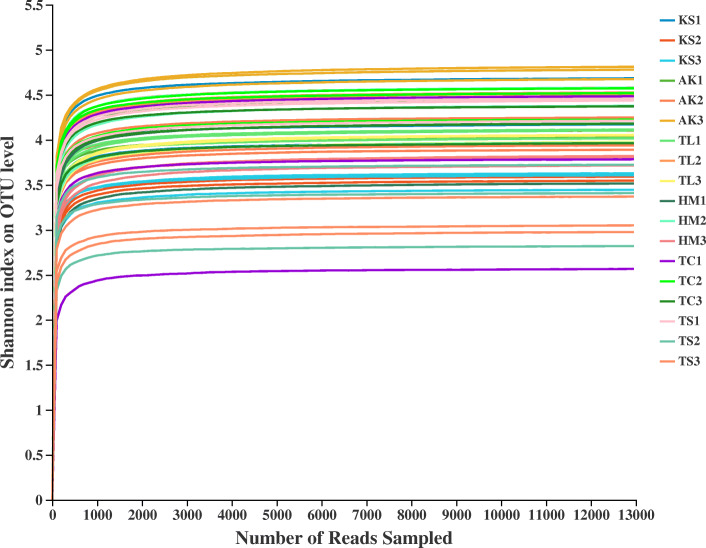
Rarefaction curves of rhizosphere soil samples. Rarefaction curves of OTUs were clustered for a dissimilarity threshold of 3%. Each sample had three replicates (Replicates are not specifically shown in the legend, but have been involved in the analysis). Sample abbreviations are same as presented in Fig. 1

After removing chimeras and sequences with low-quality reads, we obtained 1,952,961 fungal sequence reads of the D1 domain of the large subunit (LSU) rRNA gene from 54 soil samples. After removing non-yeast sequence reads, a total of 31, 948 yeast sequence reads were retained and clustered at 97% sequence similarity yielding 86 operational taxonomic units (OTUs). Rarefaction curves of yeast for all sequences plateaued, indicating that sequencing depth per sample was adequate to capture the diversity in the study sites (Fig. 2). In addition, we divided all samples into three large groups according to their geographical locations: Southern Xinjiang group (SX), Eastern Xinjiang group (EX) and Northern Xinjiang group (NX).

The observed species richness (Sobs), estimated richness (Chao1 and ACE indices) and species diversity (Shannon and Simpson indices) showed that the richness of yeast in the Northern Xinjiang was higher than that in the Southern and Eastern Xinjiang, but the diversity was significantly lower than them (*P* < 0.05) (Table 1). Based on the analysis of intergroup differences, all the values of Sobs, Chao1 and ACE indices of samples from Northern Xinjiang (NX) were the highest among the three groups, but there was no significant difference. The Shannon index was significantly higher in Southern Xinjiang (SX) and Eastern Xinjiang (EX) than in Northern Xinjiang, and Simpson index was significantly higher in Northern Xinjiang (NX) than in Southern Xinjiang (SX) and Eastern Xinjiang (EX).

**Table 1:** Alpha diversity indices of yeast in rhizosphere soil of Hami melon from different samples

sample group	Sample number	Sobs	Chao1	ACE	Shannon	Simpson
SX	KS1	17.33 ± 0.667b	23.83 ± 1.922ab	23.59 ± 1.314a	1.81 ± 0.073a	0.25 ± 0.021a
	KS2	13.67 ± 2.036b	16.33 ± 3.180b	19.21 ± 5.376a	1.64 ± 0.266a	0.32 ± 0.099a
	KS3	16.33 ± 1.202b	18.23 ± 1.623ab	21.12 ± 3.243a	2.16 ± 0.271a	0.19 ± 0.079a
	AK1	14.67 ± 2.906b	20.17 ± 3.444ab	21.16 ± 3.774a	2.17 ± 0.125a	0.14 ± 0.114a
	AK2	18.33 ± 0.882ab	22.33 ± 2.404ab	23.12 ± 2.841a	2.12 ± 0.367a	0.21 ± 0.028a
	AK3	23.00 ± 1.000a	26.15 ± 2.051a	27.46 ± 2.585a	2.02 ± 0.064a	0.23 ± 0.028a
	mean	17.22 ± 0.924	21.18 ± 1.183	22.61 ± 1.332	1.99 ± 0.091**A**	0.22 ± 0.029**B**
EX	TL1	10.67 ± 1.856b	12.42 ± 2.526c	16.83 ± 6.454b	1.95 ± 0.141bc	0.17 ± 0.030b
	TL2	12.67 ± 1.453b	13.73 ± 1.690c	14.80 ± 2.128b	1.95 ± 0.111bc	0.20 ± 0.025b
	TL3	22.00 ± 0.577a	23.07 ± 0.869b	24.59 ± 1.496b	2.31 ± 0.133ab	0.16 ± 0.022b
	HM1	13.33 ± 0.333b	14.61 ± 1.369c	18.20 ± 4.635b	1.55 ± 0.108c	0.36 ± 0.052a
	HM2	24.00 ± 1.528a	30.83 ± 2.309a	39.85 ± 7.355a	2.57 ± 0.183a	0.11 ± 0.026b
	HM3	25.00 ± 0.577a	27.50 ± 0.289ab	27.77 ± 1.002ab	1.56 ± 0.103c	0.37 ± 0.059a
	mean	17.94 ± 1.476	20.36 ± 1.832	23.67 ± 2.591	1.98 ± 0.101**A**	0.23 ± 0.028**B**
NX	TC1	18.00 ± 0.577b	18.37 ± 0.731b	19.01 ± 0.898a	1.71 ± 0.095ab	0.31 ± 0.037ab
	TC2	22.33 ± 1.202a	24.75 ± 2.126ab	24.51 ± 1.898a	1.50 ± 0.114ab	0.42 ± 0.042ab
	TC3	22.67 ± 0.333a	28.17 ± 3.321a	42.39 ± 17.034a	1.84 ± 0.105a	0.27 ± 0.040b
	TS1	22.67 ± 0.006a	23.83 ± 0.667ab	24.67 ± 0.282a	1.63 ± 0.552ab	0.32 ± 0.023ab
	TS2	19.00 ± 2.517ab	20.61 ± 2.772b	22.13 ± 3.138a	0.63 ± 0.121b	0.75 ± 0.052a
	TS3	16.67 ± 0.003b	19.63 ± 1.978b	20.34 ± 0.931a	1.06 ± 0.148ab	0.52 ± 0.068ab
	mean	20.22 ± 0.725	22.56 ± 1.103	25.51 ± 3.096	1.40 ± 0.109**B**	0.43 ± 0.043**A**

Note: Samples abbreviations are as in Fig. 1. Each sample had three replicates. Sobs index was the observed species richness, Chao1 and ACE indices were used to evaluate species richness, Shannon and Simpson indices were used to evaluate species diversity. Larger Simpson index values indicate lower species diversity. The values of mean ± SE (standard error) of three samples are shown in the table. The different lowercase letters are significantly difference within groups, the different capital letters are significantly difference among groups. (Kruskal-Wallis test, *P* < 0.05)

### Yeast community composition

**Fig. 3:**
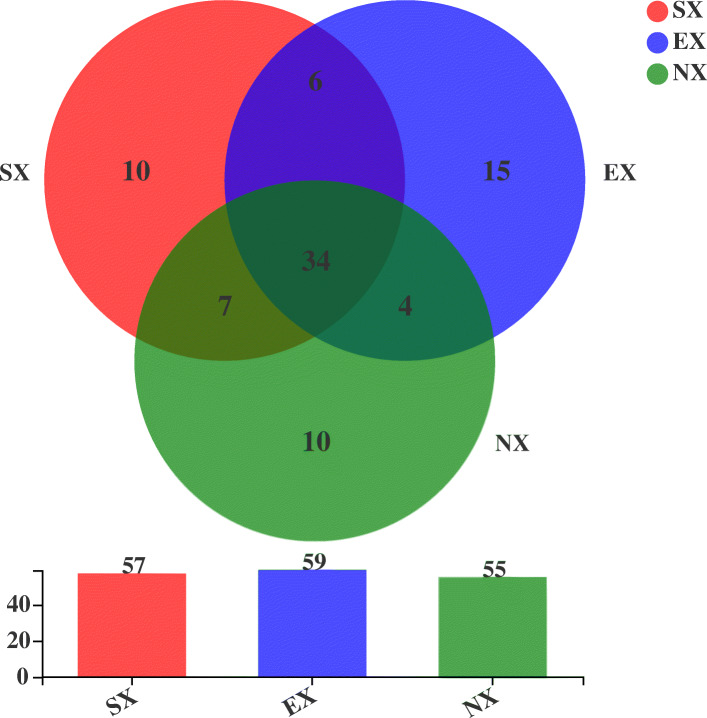
Venn diagram at the OTU level of soil samples in Southern Xinjiang (SX), Eastern Xinjiang (EX) and Northern Xinjiang (NX). Each circle with different colors in the diagram represents a group; middle core numbers represent the number of OTUs common to all groups. The shared and unique yeast OTUs were shown at a 0.03 dissimilarity distance after removing singletons

**Fig. 4:**
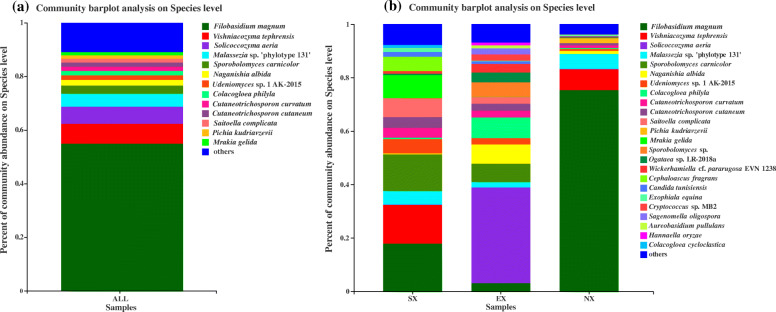
Proportion of dominant yeasts in soil samples. (**a**) all samples; (**b**) the samples in Southern Xinjiang (SX), Eastern Xinjiang (EX), and Northern Xinjiang (NX). Others indicated that species accounted for less than 1%. Sample abbreviations are same as presented in Fig. 1

The numbers of yeast sequence reads and OTUs detected in samples from the SX, EX, and NX were 4268 and 57, 5616 and 59, and 22,065 and 55, respectively. We found that 34 OTUs were shared by all three groups; OTUs species were shared between SX and EX; 41 OTUs were shared between SX and NX; 38 OTUs were shared between EX and NX (Fig. 3). We identified 86 OTUs, 59 genera, and 86 species, which belonged to Ascomycota and Basidiomycota. Ascomycota contained 45 OTUs, 27 genera, and 45 species accounting for approximately 9.6% of all yeast sequences, while Basidiomycota had 41 OTUs, 32 genera, and 41 species accounting for approximately 90.4%. These include six genera of yeast-like fungi: *Aureobasidium* (0.54%), *Microglossum* (0.15%), *Basidioascus* (0.04%), *Hormonema* (0.03%) and *Cyphellophora* (0.01%), *Tilletiopsis* (0.01%), and a total of 36 rare species (Species with less than 1% frequency of occurrence) were detected (Tables 2 and 3). The dominant genera that accounted for greater than 1% were *Filobasidium* (54.97%), *Vishniacozyma* (7.32%), *Solicoccozyma* (6.41%), *Malassezia* (5.13%), *Sporobolomyces* (4.01%), *Cutaneotrichosporon* (3.16%), *Naganishia* (2.07%), *Udeniomyces* (1.92%), *Colacogloea* (1.82%)*, Pichia* (1.54%), *Saitoella* (1.41%), and *Mrakia* (1.20%). The dominant species that accounted for greater than 1% were *Filobasidium magnum* (54.97%), *Vishniacozyma tephrensis* (7.32%), *Solicoccozyma aeria* (6.41%), *Malassezia* sp. ‘phylotype 131’ (4.89%), *Sporobolomyces carnicolor* (3.05%), *Naganishia albida* (2.07%), *Udeniomyces* sp. 1 AK-2015 (1.69%), *Colacogloea philyla* (1.66%), *Cutaneotrichosporon curvatum* (1.60%), *Cutaneotrichosporon cutaneum* (1.56%), *Saitoella complicata* (1.41%), *Pichia kudriavzevii* (1.29%), and *Mrakia gelida* (1.18%) (Tables 2, 3, Fig. 4a). The 12 dominant genera and 13 dominant species accounted for 90.96 and 89.1% of all yeast sequences, respectively. *Filobasidium magnum*, the most dominant species of all yeasts, was detected in the NX accounted for 75.36%, SX and EX having 17.90 and 3.03%, respectively; *Vishniacozyma tephrensis* was the second dominant species: SX (14.50%), EX (0.05%), and NX (7.78%). The proportion of *Solicoccozyma aeria* was 35.83, 19.25, and 0.67% in SX, EX, and NX, respectively. The most dominant species in the samples from both South (SX) and North Xinjiang (NX) was *Filobasidium magnum*, *Solicoccozyma aeria* was the most dominant species in the Eastern Xinjiang (EX) (Fig. 4b).

**Table 2:** The Ascomycetous yeasts taxa (accounted for 9.6%) in the Illumina sequencing library

No. taxon	Genus	Species	No. OTU^**d**^	Similarity%	GenBank accession^**e**^	Total^**a**^	Occurrence frequency %
1	*Pichia*	*Pichia kudriavzevii*	3076	100	MT355189	1.29%	25.93%
2	*Pichia*	*Pichia kluyveri*	2013	100	MN337254	0.22%	27.78%
3^**c**^	*Pichia*	*Pichia barkeri*	1450	77.7	KX258655	0.02%	3.70%
4^**c**^	*Pichia*	*Pichia terricola*	543	100	MN904759	0.01%	1.85%
5	*Saitoella*	*Saitoella complicata*	2383	79.1	KY109522	1.41%	74.07%
6	*Wickerhamiella*	*Wickerhamiella* cf. *pararugosa* EVN 1238	804	98.1	FR853155	0.81%	68.525
7	*Wickerhamiella*	*Wickerhamiella pararugosa*	4348	94.8	MH545933	0.05%	12.96%
8	*Ogataea*	*Ogataea* sp. *LR-*2018a	4165	94.7	KY971657	0.91%	55.56%
9^**c**^	*Cephaloascus*	*Cephaloascus fragrans*	2323	99.6	NG 063972	0.72%	1.85%
10	*Candida*	*Candida tunisiensis*	2493	95.2	NG_060831	0.56%	68.52%
11	*Candida*	*Candida ethanolica*	1626	100	MK660230	0.19%	24.07%
12	*Candida*	*Candida tropicalis*	4288	100	CP047875	0.16%	33.33%
13	*Candida*	*Candida* sp. *UFMG DC* 166	1518	94.7	KF695404	0.12%	29.63%
14	*Candida*	*Candida rugopelliculosa*	224	81.9	KY106729	0.03%	11.11%
15^**c**^	*Candida*	*Candida* sp.	1683	78.9	KY385325	0.02%	3.70%
16^**c**^	*Candida*	*Candida boidinii*	1172	100	MN058032	0.01%	1.85%
17	*Geotrichum*	*Geotrichum* sp.	4031	100	MT312851	0.68%	38.89%
18^**c**^	*Geotrichum*	*Geotrichum* sp. YM24346	3180	79.7	HQ689675	0.02%	7.41%
19	*Aureobasidium*^**b**^	*Aureobasidium pullulans*	4882	100	MT448852	0.36%	18.52%
20	*Aureobasidium*	*Aureobasidium* sp.	3867	98.2	KX263043	0.18%	14.81%
21	*Exophiala*	*Exophiala equina*	2468	99.6	MT453276	0.43%	57.41%
22	*Sagenomella*	*Sagenomella oligospora*	4653	100	LT633931	0.39%	11.11%
23	*Microglossum*^**b**^	*Microglossum aff. Nudipes* MT-2017	4482	79.4	KX382836	0.13%	22.22%
24^**c**^	*Microglossum*	*Microglossum viride*	630	79.1	AY789337	0.02%	5.56%
25	*Starmerella*	*Starmerella bacillaris*	4665	100	MN904789	0.08%	14.81%
26^**c**^	*Starmerella*	*Starmerella bombi*	2995	100	LT631805	0.05%	3.70%
27^**c**^	*Starmerella*	*Starmerella lactis-condensi*	2845	100	MK513740	0.01%	1.85%
28	*Cyberlindnera*	*Cyberlindnera jadinii*	1070	100	NG 056278	0.10%	35.19%
29^**c**^	*Cyberlindnera*	*Cyberlindnera fabianii*	1444	100	MK392110	0.04%	1.85%
30	*Saccharomyces*	*Saccharomyces cerevisiae*	2462	100	MT420738	0.13%	24.07%
31^**c**^	*Hanseniaspora*	*Hanseniaspora opuntiae*	1168	100	MN904783	0.05%	9.26%
32	*Kazachstania*	*Kazachstania humilis*	297	93.1	EU149661	0.04%	11.11%
33^**c**^	*Schizosaccharomyces*	*Schizosaccharomyces japonicus*	4897	74.9	MK690482	0.04%	5.56%
34	*Torulaspora*	*Torulaspora delbrueckii*	1536	100	MT449110	0.04%	11.11%
35^**c**^	*Saturnispora*	*Saturnispora zaruensis*	4590	100	KY109556	0.02%	9.26%
36^**c**^	*Saturnispora*	*Saturnispora diversa*	1530	100	MH892856	0.01%	3.70%
37^**c**^	*Hormonema*^**b**^	*Hormonema carpetanum*	4014	100	MF611880	0.03%	9.26%
38^**c**^	*Meyerozyma*	*Meyerozyma caribbica*	4075	100	MH545919	0.03%	7.41%
39^**c**^	*Wickerhamomyces*	*Wickerhamomyces pijperi*	3312	100	KY630162	0.01%	1.85%
40^**c**^	*Wickerhamomyce*	*Wickerhamomyces hampshirensis*	2398	100	KY110121	0.01%	1.85%
41^**c**^	*Nakazawaea*	*Nakazawaea ishiwadae*	2385	100	MN174047	0.02%	1.85%
42^**c**^	*Eremascus*	*Eremascus albus*	550	95.6	LT964976	0.02%	7.41%
43^**c**^	*Cyphellophora*^**b**^	*Cyphellophora* sp. JCM *28586*	2971	100	LC134275	0.01%	5.56%
44^**c**^	*Metschnikowia*	*Metschnikowia* sp. JJW-2*009a*	3027	88.7	FJ794937	0.01%	3.70%
45^**c**^	*Yamadazyma*	*[Candida] amphicis*	2393	100	LC435604	0.01%	1.85%

Note: ^**a**^ Percent of sequences in Illumina sequencing library, ^**b**^ yeast-like fungi, ^**c**^ rare yeasts of the species (species with an occurrence frequency of less than 10% in all samples), ^**d**^ OTU numbers obtained based on 97% similarity clustering, ^**e**^ Accession numbers of the closest homologous sequences in GenBank

**Table 3:** The Basidiomycetes yeasts taxa (accounted for 90.4%) in the Illumina sequencing library

No. taxon	Genus	Species	No. OTU^**d**^	Similarity%	GenBank accession^**e**^	Total^**a**^	Occurrence frequency %
1	*Filobasidium*	*Filobasidium magnum*	4396	100	MG367281	54.97%	74.07%
2	*Vishniacozyma*	*Vishniacozyma tephrensis*	4287	100	LC515100	7.32%	70.37%
3	*Solicoccozyma*	*Solicoccozyma aeria*	4890	100	MT408831	6.41%	29.63%
4	*Malassezia*	*Malassezia* sp. ‘phylotype 131’	1167	94	MF983553	4.89%	61.11%
5	*Malassezia*	*Malassezia restricta*	1566	100	CP033152	0.17%	48.15%
6	*Malassezia*	*Malassezia globosa*	3413	100	CP046435	0.07%	14.81%
7	*Sporobolomyces*	*Sporobolomyces carnicolor*	731	74.5	LC430208	3.05%	61.11%
8^**c**^	*Sporobolomyces*	*Sporobolomyces* sp.	4656	100	MN180193	0.96%	1.85%
9	*Cutaneotrichosporon*	*Cutaneotrichosporon curvatum*	573	100	KY107311	1.60%	96.30%
10	*Cutaneotrichosporon*	*Cutaneotrichosporon cutaneum*	232	100	KF488805	1.56%	100%
11	*Naganishia*	*Naganishia albida*	4479	100	MT448828	2.07%	50.26%
12	*Udeniomyces*	*Udeniomyces* sp. 1 AK-2015	4925	100	LN871179	1.69%	42.59%
13	*Udeniomyces*	*Udeniomyces* sp.	2770	100	MH697744.1	0.23%	18.52%
14	*Colacogloea*	*Colacogloea philyla*	565	75.3	KY106944	1.66%	55.56%
15^**c**^	*Colacogloea*	*Colacogloea cycloclastica*	2072	86.9	KY106939	0.16%	5.56%
16	*Mrakia*	*Mrakia gelida*	3246	100	MT133537	1.18%	40.74%
17^**c**^	*Mrakia*	*Mrakia* sp.	4600	80.8	MT505691	0.02%	3.70%
18	*Cryptococcus*	*Cryptococcus* sp. MB2	570	75.8	KF830205	0.42%	11.11%
19^**c**^	*Cryptococcus*	*Cryptococcus* sp.	3601	85.9	MN299301	0.01%	3.70%
20	*Sporidiobolus*	*Sporidiobolus metaroseus*	2333	100	MN075296	0.42%	22.22%
21	*Vanrija*	*Vanrija humicola*	2632	79.6	KP294523	0.35%	12.96%
22^**c**^	*Vanrija*	*Vanrija nantouana*	172	93.8	NG 058428	0.01%	1.85%
23	*Hannaella*	*Hannaella oryzae*	1375	75.1	LC428182	0.19%	20.37%
24	*Papiliotrema*	*Papiliotrema* sp.	3147	100	MN609790	0.18%	29.63%
25	*Kockovaella*	*Kockovaella* sp.	378	78	MT252009	0.11%	12.96%
26	*Spencerozyma*	*Spencerozyma* sp.	4395	73.7	MK131326	0.11%	11.11%
27	*Rhodotorula*	*Rhodotorula* sp.	2893	100	MT533781	0.09%	18.52%
28	*Leucosporidium*	*Leucosporidium* sp.	916	99.6	MK271689	0.08%	20.37%
29	*Tausonia*	*Tausonia* sp. KBP 4496	4243	83.1	LN871177	0.08%	18.52%
30	*Sympodiomycopsis*	*Sympodiomycopsis* sp.	4986	79.8	KY594009	0.08%	14.81%
31	*Sterigmatomyces*	*Sterigmatomyces elviae*	1557	77.1	KY109789	0.06%	11.11%
32^**c**^	*Piskurozyma*	*Piskurozyma* sp.	368	82.8	MT470199	0.06%	7.41%
33	*Basidioascus*^**b**^	*Basidioascus persicus*	5003	100	KC751416	0.04%	11.11%
34	*Dioszegia*	*Dioszegia* sp.	3967	100	MK050296	0.03%	11.11%
35^**c**^	*Occultifur*	*Occultifur kilbournensis*	2106	99.6	NG 060322	0.02%	9.26%
36^**c**^	*Apiotrichum*	*Apiotrichum montevideense*	1009	100	MN872920	0.02%	1.85%
37^**c**^	*Kurtzmanomyces*	*Kurtzmanomyces nectairei*	4195	98.2	KY108195	0.02%	5.56%
38^**c**^	*Cystofilobasidium*	*Cystofilobasidium infirmominiatum*	4574	100	MF927610	0.02%	7.41%
39^**c**^	*Cystobasidium*	*Cystobasidium lysinophilum*	2642	100	LC203672	0.01%	7.41%
40^**c**^	*Moniliella*	*Moniliella byzovii*	3043	80.8	NG 060289	0.01%	1.85%
41^**c**^	*Tilletiopsis*^**b**^	*Tilletiopsis washingtonensis*	4097	100	MH868275	0.01%	1.85%

Note: ^**a**^ Percent of sequences in Illumina sequencing library, ^**b**^ yeast-like fungi, ^**c**^ rare yeasts of the species (species with an occurrence frequency of less than 10% in all samples), ^**d**^ OTU numbers obtained based on 97% similarity clustering, ^**e**^ Accession numbers of the closest homologous sequences in GenBank

**Fig. 5:**
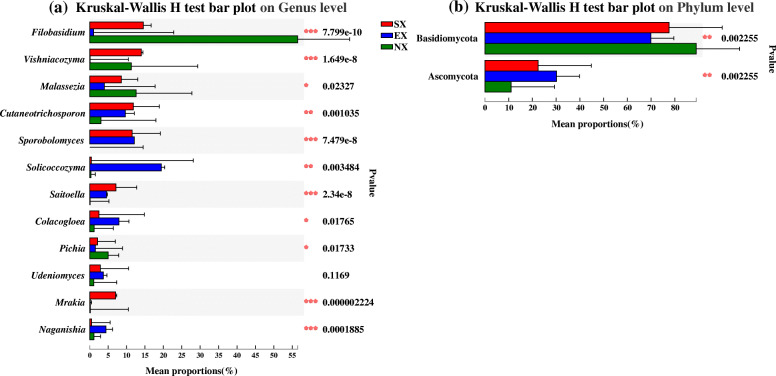
Species difference analysis of the samples in Southern Xinjiang (SX), Eastern Xinjiang (EX) and Northern Xinjiang (NX): (**a**) at the genus level; (**b**) at the phylum level. The y-axis represents the classification levels of species, and the x-axis represents the percentage of species average relative abundance in each sample group. The red, blue, and green columns represent the average results in the SX, EX, and NX soil samples, respectively. The Kruskal-Wallis rank-sum test was used to show significant differences (*: 0.01 < *P* < = 0.05, **: 0.001 < *P* < = 0.01, ***: *P* < = 0.001)

The results of the analysis of species differences between groups based on the phylum level showed that the proportion of Basidiomycota was significantly higher than that of Ascomycota among all soil samples from three groups, Basidiomycota was considered to be the dominant phylum. The proportion of Ascomycota in EX was significantly higher than that in SX and NX (*P* < 0.05) (Fig. 5b). At the genus level, there were 11 dominant genera with significant differences in relative abundance (*P* < 0.05) among SX, EX, and NX, except for *Udeniomyces*. *Filobasidium* and *Vishniacozyma* were mainly present in the samples from Southern and Northern Xinjiang; *Sporobolomyces*, *Cutaneotrichosporon* and *Saitoella* were detected mainly in samples from the Southern and Eastern Xinjiang; *Solicoccozyma* and *Mrakia* are found mainly in the Eastern and Southern Xinjiang, respectively (Fig. 5a).

### Relationship between yeast communities in samples from different regions

**Fig. 6:**
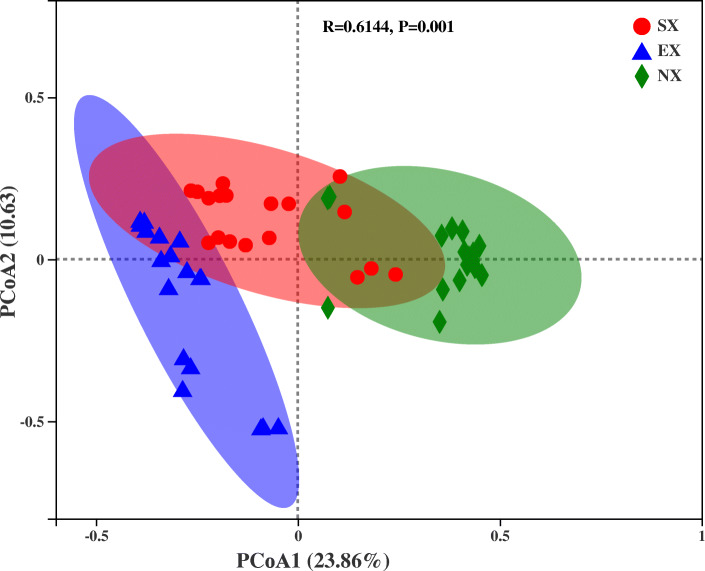
Principal Coordinates analysis (PCoA) based on Bray-Curtis distance method at the OTU level. Red circles, blue triangles and green diamonds represent samples from SX, EX, and NX, respectively. Each sample had three replicates

We performed ordination by PCoA at the OTU level to reveal similarities or differences in community composition among grouped samples (Fig. 6). The first principal coordinates axis (PCoA1) and the second principal coordinates axis (PCoA2) alone explained 23.86 and 10.63% of the variance, respectively. PCoA1 has relatively small eigenvalues, capturing less than 50% of the variation in the input data, and therefore is not considered a very successful PCoA. However, R value (0.6144) greater than 0 indicates that the difference between sample groups is greater than the differences within groups and that the difference is significant (*P* < 0.05). Overall, most samples from each group were clustered together, with only a slight overlapping among the samples from the three groups on the score plots, indicating significant differences in community composition between groups. For inter-groups, the SX and EX were more similar in community composition, and this result can also be observed visually in the box plot of PCoA (Fig. S1).

### Relationship between yeast community structure and environmental factors

**Fig. 7:**
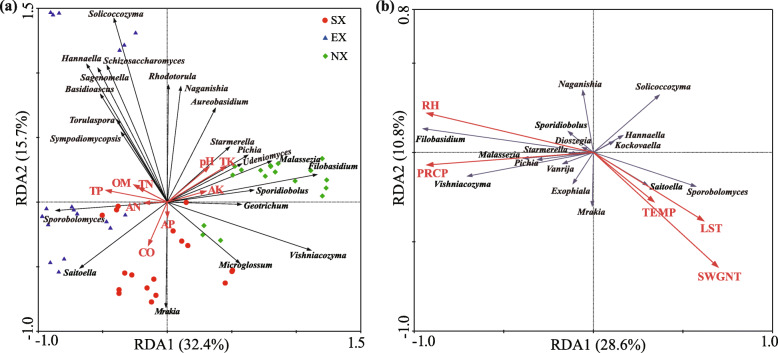
Redundancy analysis (RDA) of (**a**) the correlation between the yeast community and soil physicochemical properties in all samples from three regions of Xinjiang, and (**b**) the correlation between the yeast community and climate factors at the genus level. Red, blue, and green symbols represent samples from SX, EX, and NX, respectively. Red and black arrows represent the soil parameters and genera, respectively. Soil physicochemical properties: pH, Conductivity (CO), Organic matter (OM), Total nitrogen (TN), Total phosphorus (TP), Total potassium (TK), Available nitrogen (AN), Available phosphorus (AP), Available potassium (AK). Climate factors: Average annual precipitation (PRECTP), Average annual temperature (TEMP), Average annual land surface temperature (LST), Average annual relative humidity (RH), The annual average net solar radiation intensity received by the earth’s surface (SWGNT)

The statistical results of soil physicochemical properties showed that the values of conductivity (CO) in the Southern Xinjiang (SX), the levels of organic matter (OM) and total phosphorus (TP) in the Eastern Xinjiang (EX), the pH, total potassium (TK) and available potassium (AK) values in the Northern Xinjiang (NX) are significantly higher than in the other two regions (*P* < 0.05). Available nitrogen (AN) content of NX was significantly lower than SX and EX (*P* < 0.05) (Table 4). Redundancy analysis based on yeast genera and soil physicochemical properties in soil samples from different regions showed that the first and second RDA components explained 43.6% of the total variation (Fig. 7a). CO, TP and TK were significantly associated with the yeast community (*P* < 0.05), and mainly influenced the distribution of samples in the Southern (SX), Eastern (EX) and Northern Xinjiang (NX), respectively. These results suggest a correlation between the yeast community and soil physicochemical properties, particularly total phosphorus (TP) content in the soil. The F-ratio and *P* values for each soil factor are shown in Table S1.

**Table 4:** The physicochemical properties of rhizosphere soil of Hami Melon in different regions

sample group	Sample number	pH	CO (mS/cm)	OM (g/kg)	TN (g/kg)	TP (g/kg)	TK (g/kg)	AN (mg/kg)	AP (mg/kg)	AK (mg/kg)
SX	KS1	7.99 ± 0.007ab	1.15 ± 1.732a	7.53 ± 0.087b	0.57 ± 0.002b	0.79 ± 0.011b	18.24 ± 0.168a	42.20 ± 0.064b	18.80 ± 0.183b	96.47 ± 2.636ab
	KS2	7.85 ± 0.007ab	1.11 ± 1.732ab	10.07 ± 0.030ab	1.03 ± 0.241ab	0.94 ± 0.005ab	18.25 ± 0.179a	71.72 ± 0.356ab	35.81 ± 0.206ab	103.73 ± 2.008ab
	KS3	7.92 ± 0.003ab	1.08 ± 2.963ab	10.08 ± 0.010ab	0.77 ± 0.013ab	0.82 ± 0.004ab	18.12 ± 0.089a	62.66 ± 0.384ab	25.25 ± 3.153ab	102.77 ± 0.318ab
	AK1	8.16 ± 0.007a	0.47 ± 5.840ab	12.89 ± 0.079ab	0.97 ± 0.011ab	0.83 ± 0.012ab	19.48 ± 0.130a	140.24 ± 0.670ab	31.50 ± 0.369ab	304.60 ± 1.484a
	AK2	8.04 ± 0.003ab	0.45 ± 0.333b	13.77 ± 0.056ab	0.87 ± 0.010ab	0.99 ± 0.011ab	19.12 ± 0.372a	54.54 ± 0.557ab	57.25 ± 9.575ab	60.17 ± 0.517b
	AK3	7.51 ± 0.003b	0.65 ± 3.464ab	18.01 ± 0.194a	1.43 ± 0.030a	1.25 ± 0.00780a	19.79 ± 0.081a	289.71 ± 4.556a	158.23 ± 1.295a	180.57 ± 0.696ab
	mean	7.91 ± 0.050**B**	0.82 ± 0.074**A**	12.06 ± 0.814**AB**	0.94 ± 0.073	0.94 ± 0.039**B**	18.83 ± 0.174**B**	110.18 ± 20.918**A**	54.47 ± 11.70846	141.38 ± 19.735**B**
EX	TL1	8.17 ± 0.003ab	0.17 ± 0.700ab	9.27 ± 0.240b	0.57 ± 0.010b	1.15 ± 0.013ab	19.19 ± 0.148ab	56.64 ± 0.819b	35.37 ± 0.182ab	136.60 ± 0.874ab
	TL2	7.96 ± 0.003ab	0.37 ± 2.186ab	11.09 ± 0.042ab	0.76 ± 0.011ab	1.13 ± 0.005ab	18.76 ± 0.284ab	75.12 ± 0.508ab	51.63 ± 0.518a	180.37 ± 1.812ab
	TL3	8.24 ± 0.009ab	0.19 ± 2.881ab	9.11 ± 0.027b	0.68 ± 0.014ab	0.90 ± 0.008b	22.88 ± 0.099ab	57.87 ± 0.407ab	16.83 ± 0.305b	172.87 ± 0.982ab
	HM1	8.24 ± 0.012ab	0.19 ± 0.657ab	22.20 ± 0.301ab	1.38 ± 0.011ab	1.06 ± 0.001ab	25.68 ± 0.164a	248.86 ± 4.012ab	25.10 ± 0.432ab	246.87 ± 0.617a
	HM2	7.76 ± 0.003b	1.10 ± 2.517a	43.73 ± 0.038a	2.40 ± 0.011a	1.02 ± 0.003ab	13.46 ± 0.135b	2795.73 ± 10.3201a	46.61 ± 0.143ab	240.07 ± 3.223ab
	HM3	8.25 ± 0.010a	0.09 ± 1.594b	18.72 ± 0.185ab	1.22 ± 0.036ab	1.22 ± 0.020a	21.55 ± 0.093ab	87.33 ± 0.154ab	40.27 ± 0.296ab	71.30 ± 1.386b
	mean	8.10 ± 0.044**B**	0.35 ± 0.083**B**	19.02 ± 2.933**A**	0.17 ± 0.151	1.08 ± 0.025**A**	20.25 ± 0.928**B**	553.59 ± 243.73**A**	35.97 ± 2.90898	174.68 ± 14.584**B**
NX	TC1	8.22 ± 0.003b	0.30 ± 1.764a	13.79 ± 0.165a	1.00 ± 0.007ab	0.91 ± 0.005a	25.41 ± 0.081ab	65.37 ± 0.202ab	30.77 ± 0.157ab	402.27 ± 2.204ab
	TC2	8.23 ± 0.003ab	0.24 ± 1.202ab	13.79 ± 0.152a	1.05 ± 0.005a	0.88 ± 0.009ab	25.47 ± 0.215ab	73.36 ± 0.511a	40.33 ± 0.395a	414.30 ± 1.217a
	TC3	8.46 ± 0.006ab	0.19 ± 3.075ab	9.44 ± 0.109ab	0.66 ± 0.007ab	0.74 ± 0.007ab	24.69 ± 0.098b	40.21 ± 0.293ab	29.89 ± 0.470ab	249.27 ± 1.157ab
	TS1	8.65 ± 0.012a	0.16 ± 7.169b	6.11 ± 0.032ab	0.39 ± 0.004ab	0.57 ± 0.002b	25.20 ± 0.174ab	29.40 ± 0.161b	17.47 ± 0.170b	170.87 ± 1.753ab
	TS2	8.56 ± 0.003ab	0.25 ± 0.882ab	5.23 ± 0.094b	0.35 ± 0.009b	0.60 ± 0.005ab	26.46 ± 0.135a	43.34 ± 0.136ab	26.85 ± 0.248ab	149.17 ± 1.525b
	TS3	8.32 ± 0.038ab	0.18 ± 0.809ab	10.35 ± 0.532ab	0.73 ± 0.017ab	0.69 ± 0.007ab	24.72 ± 0.078b	49.92 ± 0.202ab	20.35 ± 0.078ab	251.67 ± 0.940ab
	mean	8.41 ± 0.039**A**	0.22 ± 0.012**B**	9.76 ± 0.808**B**	0.70 ± 0.065	0.73 ± 0.031**C**	25.32 ± 0.151**A**	50.27 ± 3.634**B**	27.61 ± 1.810	272.92 ± 24.949**A**

Note: Sample abbreviations are as in Fig. 1. Each sample had three replicates. Soil physicochemical properties: pH, Conductivity (*CO*), Organic matter (*OM*), Total nitrogen (*TN*), Total phosphorus (*TP*), Total potassium (*TK*), Available nitrogen (*AN*), Available phosphorus (*AP*), Available potassium (*AK*). The values of mean ± SE (standard error) of three samples are shown in the table. The different lowercase letters are significantly difference within groups, the different capital letters are significantly difference among groups. (Kruskal-Wallis test, *P* < 0.05)

The results of the analysis of climatic factors at the sampling sites in different regions show that the average annual precipitation (PRCP) and relative humidity (RH) were significantly higher in Northern Xinjiang (NX) than in Southern (SX) and Eastern Xinjiang (EX), and the lowest in Eastern Xinjiang (EX) (*P* < 0.05). The average annual temperature (TEMP), land surface temperature (LST) and net solar radiation intensity (SWGNT) in Southern (SX) and Eastern Xinjiang (NX) are higher than in Northern Xinjiang, but there is no significant difference (Table 5). Redundancy analysis (RDA) of the correlation between the yeast community and climate factors showed that the first and second RDA components explained 39.4% of the total variation. PRCP, RH and SWGNT were the climatic factors that have significant effects on the distribution of yeast communities (*P* < 0.05) (Fig. 7b). PRCP and RH were negatively correlated with SWGNT, and were positively correlated with *Filobasidium* and *Vishniacozyma* but negatively correlated with *Solicoccozyma.* SWGNT was positively correlated with *Sporobolomyces*, *Cutaneotrichosporon* and *Saitoella.* The F-ratio and *P* values for each climate factor are shown in Table S2.

**Table 5:** The climate factors for 2019 at different sampling locations

sample group	Sample number	PRCP (mm)	TEMP (°C)	LST (°C)	RH (%)	SWGNT (W/m^**2**^)
SX	KS1	5.12 ± 1.627	13.28 ± 3.255	14.75 ± 3.877	30.64 ± 2.542ab	154.6 ± 17.44
	KS2	5.42 ± 1.815	13.18 ± 3.244	14.48 ± 3.849	31.27 ± 2.645ab	155.0 ± 18.29
	KS3	2.97 ± 4.357	13.44 ± 3.176	14.99 ± 3.722	29.66 ± 2.553b	159.2 ± 17.42
	AK1	10.18 ± 2.909	10.15 ± 3.348	8.28 ± 3.622	41.77 ± 2.672a	144.1 ± 15.97
	AK2	8.09 ± 2.983	12.37 ± 3.382	13.26 ± 4.108	34.41 ± 3.109ab	144.3 ± 16.80
	AK3	8.23 ± 2.817	12.55 ± 3.391	13.32 ± 4.125	34.27 ± 3.149ab	141.2 ± 16.08
	mean	6.67 ± 0.963**B**	12.50 ± 1.306	13.18 ± 1.554	33.67 ± 1.198**B**	149.7.1 ± 6.75
EX	TL1	1.42 ± 0.293	17.01 ± 4.311	13.47 ± 4.363	30.42 ± 3.457	159.6 ± 19.71
	TL2	1.11 ± 0.287	14.68 ± 4.054	16.33 ± 4.471	27.55 ± 3.086	157.2 ± 18.89
	TL3	1.05 ± 0.308	17.84 ± 4.350	14.00 ± 4.385	29.75 ± 3.325	157.3 ± 19.59
	HM1	2.07 ± 0.840	11.01 ± 3.947	13.37 ± 4.407	26.93 ± 2.452	169.1 ± 20.34
	HM2	2.71 ± 1.168	4.66 ± 3.405	11.70 ± 4.409	28.97 ± 2.645	172.0 ± 19.67
	HM3	2.15 ± 0.949	7.85 ± 3.716	12.74 ± 4.395	27.83 ± 2.571	170.4 ± 20.48
	mean	1.75 ± 0.298**C**	12.17 ± 1.666	13.60 ± 1.742	28.57 ± 1.170**C**	164.3 ± 7.82
NX	TC1	17.94 ± 5.346	11.08 ± 4.158	10.90 ± 4.428	45.40 ± 4.877	145.5 ± 20.05
	TC2	15.77 ± 4.384	10.28 ± 4.221	9.98 ± 4.489	46.21 ± 5.206	134.8 ± 19.72
	TC3	14.55 ± 3.773	10.65 ± 4.227	10.42 ± 4.639	46.69 ± 5.650	138.5 ± 20.48
	TS1	17.32 ± 3.667	10.63 ± 4.176	10.23 ± 4.340	47.41 ± 4.808	148.6 ± 20.60
	TS2	10.58 ± 2.150	10.28 ± 4.521	11.40 ± 4.651	46.06 ± 5.678	147.7 ± 21.02
	TS3	17.30 ± 3.927	8.79 ± 4.274	9.86 ± 4.131	46.84 ± 3.999	148.8 ± 20.37
	mean	15.58 ± 1.599**A**	10.28 ± 1.681	10.47 ± 1.753	46.44 ± 1.997**A**	144.0 ± 8.05

Note: Sample abbreviations are as in Fig. 1. Climate factors: Average annual precipitation (*PRCP*), Average annual temperature (*TEMP*), Average annual land surface temperature (*LST*), Average annual relative humidity (*RH*), The annual average net solar radiation intensity received by the earth’s surface (*SWGNT*). The values of mean ± SE (standard error) of twelve months are shown in the table. The different lowercase letters are significantly difference within groups, the different capital letters are significantly difference among groups. (Kruskal-Wallis test, *P* < 0.05)

## Discussion

### Yeast diversity in rhizosphere soils of Hami melon orchards

A total of two phyla, 59 genera and 86 species of yeasts were detected based on high-throughput sequencing technology in this study (Tables 2 and 3). Using a combination of MALDI-TOF MS and rDNA sequencing, previous scholars identified a total of 60 yeast species from 200 soil samples of five fruit trees (apple, pear, plum, peach and apricot) from two locations in southwest Slovakia [27]. Moreover, only 16 species of yeast were detected in 493 samples of Cameroon-based agricultural soil from nine locations using the culture-dependent method [28]. This indicates that there are rich yeast resources with a relatively high yeast diversity in the rhizosphere soil of Xinjiang Hami melon. On the one hand, the high level of yeast diversity may be related to the high sugar content of Xinjiang Hami melon (15–18%) compared to other fruits and vegetables such as watermelon (7–11%), tomatoes (7–10%) and apples (10–14.2%) [29–32]. On the other hand, tillage practices also influence the diversity and abundance of soil microorganisms, for example, crop rotation is more conducive to the accumulation of mycorrhizal species than continuous cropping [33–35]. During our sampling, we learned that crop rotation is commonly used in Xinjiang Hami melon fields to avoid pests and soil micronutrient deficiencies [36]. Furthermore, epiphytic yeasts from the surfaces of various plant species entering the soil with humus during crop rotation may also further increase soil yeast diversity in Hami melon orchards [37]. This is because, the Basidiomycete genera *Vishniacozyma*, *Sporobolomyces, Kockovaella, Rhodotorula* and *Cystobasidium* in this study were usually isolated from plant surfaces in most studies [33, 37].

Ascomycota was the more diverse phylum, but its abundance was much lower than that of Basidiomycetes (Tables 2, 3 and Fig. 5b), which challenged the traditional view that Ascomycetous yeasts were generally more frequent and abundant in agricultural soils, orchards, and grasslands [38, 39]. Other studies revealed that Basidiomycetes were dominant in forest soils [24, 38, 40]. This may be the result of differences in research methods. Although in this study Basidiomycetes were found to have a greater advantage by high-throughput sequencing analysis, the opposite result may be obtained by culture-dependent method: since Ascomycetous have the advantage of faster growth than Basidiomycetes yeasts during culture [33]. In fact, the conclusion obtained in this study is not an isolated case, as there are previous studies on yeast in citrus orchards soil in which Basidiomycetes yeast is also dominant [41]. In addition, another study has shown that the rhizosphere of maize seedlings (20 d) was harbored only by yeasts of the phylum “Ascomycota”, whereas the rhizosphere of senescent plants (90 d) was inhabited by basidiomycetous yeasts [42]. The samples collected in this study were from rhizosphere soil at the ripening stage of Hami melon, which may also account for the higher abundance of basidiomycetous yeasts.

The rare yeast found in this study accounted for approximately 41.86% of the yeast species in all soil samples of Hami melon (Tables 2, 3), a value within the range of the proportion of rare yeast isolated by other studies from fruit trees, forests, grasslands, and shrub soils [27, 43, 44]. *Cutaneotrichosporon cutaneum* and *Cutaneotrichosporon curvatum* were also found in most samples, suggesting that the genus *Cutaneotrichosporon* may be resident yeast in the rhizosphere soil of Hami melon orchards. A strain of *Cutaneotrichosporon cutaneum* was found to be highly tolerant to tetracycline antibiotics, chloramphenicol, copper and zinc ions, and to degrade oxytetracycline with high efficiency, which could play a positive role in the prevention of environmental antibiotic contamination [45]. *Cutaneotrichosporon curvatus* belongs to the oleaginous yeast, which can be used as a biofuel [3, 9, 46]. So, the rhizosphere soil of Hami melon orchards is a potential bioprospecting soil for oleaginous yeasts for biodiesel production. *Filobasidium magnum*, *Naganishia albida* and *Mrakia gelida* belong to three of the dominant species in this study. The first two are capable of producing extracellular polymerases that contribute to the stabilisation of the soil structure and the last can be used in the food industry for brewing low-alcohol beer [47, 48]. In addition, we also detected the pathogenic fungus *Malassezia restricta*, which causes aggravate atopic dermatitis (AD), and *Tilletiopsis washingtonensis*, which produces hydrolases and antifungal compounds that can be used as antagonists of powdery mildew fungi in agricultural production, but their abundance was low [49, 50].

### Drivers affecting differences in yeast diversity and community structure in different regions

Alpha diversity analysis revealed differences in species richness and diversity among the three regions (Table 1), and the structure of the rhizosphere soil yeast communities of Hami melon also showed geographical differences among the three regions, with the Eastern and Southern Xinjiang being more similar (Fig. 6). We hypothesize that differences in soil physicochemical properties and environmental climate may be the main drivers of the differences in yeast community composition among the three regions of Xinjiang. The Changji and Shihezi areas are in the temperate grey-brown desert soil grey desert soil zone, while Tarim Basin and the Turpan-Hami Basin are in the Warm temperate brown desert soil zone [51]. Soil physicochemical properties analysis also revealed significant differences in soil type between the three areas (Table 4), and RDA analysis showed that electrical conductivity (CO), total phosphorus (TP) and total potassium (TK) were significantly correlated with the yeast community (Fig. 7a). These three factors were positively correlated with the dominant yeast genus in the Southern, Eastern, and Northern Xinjiang, respectively. The strongest correlation between total phosphorus (TP) and the yeast community may be due to the fact that phosphorus is a key element in the nutrient conversion between plants and yeast [52–54].

The meteorological data show that radiation intensity and precipitation considerably differed among three regions in Xinjiang (Table 5). And the results of redundancy analysis showed that the average annual precipitation (PRCP), relative humidity (RH) and net solar radiation intensity (SWGNT) were significantly correlated with yeast communities (Fig. 7b). There are differences in radiation levels in the Southern (293–322 KJ/cm^2^ per year), Eastern (304–307 KJ/cm^2^ per year) and Northern Xinjiang (262–277 KJ/cm^2^ per year), with the former two being hotter and more evaporative than the latter [55]. However, the precipitation situation was the opposite. Due to the influence of the warm and humid air currents from Siberia, the climate in Northern Xinjiang is relatively humid, with a little more rainfall; while Southern and Eastern Xinjiang is surrounded by mountains and is characterised by an arid climate with little rainfall; the more complex topography of Eastern Xinjiang creates a variety of habitat types [56, 57]. Additionally, previous study has shown that the abundance of yeast in soil is positively correlated with soil water content [39]. The high proportion of yeast sequence reads in this region and the fact that *Filobasidium magnum*, which is often isolated from wetter habitats and is the dominant species, was also isolated in the Northern Xinjiang and exists as a dominant species confirm the relatively wetter climate of the Northern Xinjiang [58, 59]. The higher precipitation and relative humidity of soils in the Northern border may have led to a slower decomposition of organic matter in the soil, and *Filobasidium magnum* is able to degrade or transform various organic compounds [4]. On the contrary, the most dominant species in the genus *Solicoccozyma*, *Solicoccozyma aeria*, has a preference for arid environments, mainly in the Eastern Xinjiang [52]. Combining the above information, *Filobasidium magnum* and *Solicoccozyma aeria* have the potential to serve as indicator species of ambient humidity. In addition, the Southern and Eastern Xinjiang have a high diversity of yeasts, probably due to the high level of environmental heterogeneity in Southern and Eastern Xinjiang facilitates the generation of genetic mutations and the accumulation of genetic variation in yeast [60]. Furthermore, the high quality and strong landrace of Hami melon in Southern and Eastern Xinjiang also reflect the good interplay between rhizosphere yeast community and plants [15].

## Conclusions

Our results showed that yeast resources were abundant in the soil of Hami melon orchards, and there were noticeable differences in yeast diversity and community structure among Southern, Eastern, and Northern Xinjiang. The results of this study provided interesting insights into the relationship between the yeast composition of rhizosphere soil in Hami melon orchards and their geographic regions. The results also demonstrated that both conductivity (CO), total phosphorus (TP) and Total potassium (TK) in soil factors and the average annual precipitation (PRCP), relative humidity (RH) and net solar radiation intensity (SWGNT) in climate factors have an influence on yeast community structure. The results of this study will provide a theoretical basis for better exploitation of soil yeast resources and understanding of their adaptive mechanisms.

## Methods

### Study sites and sampling

We collected rhizosphere soil samples from Hami melon orchards from six different areas within three big regions of Xinjiang between July and August 2019. Study sites included the Kashgar (35°20′ - 40°18′ N and 73°20′ - 79°57 E) and Aksu (39°30′ - 42°41′ N and 78°03′ - 84°07 E) Prefecture (SX, Southern Xinjiang), the Turpan (41°12′ - 43°40′ N and 87°16′ - 91°55 E) and Hami (40°52′ - 45°05′ N and 91°06′ - 96°23 E) Prefecture (EX, Eastern Xinjiang), the Changji (43°20′ - 45°00′ N and 85°17′ - 91°32 E) and Shihezi (43°20′ - 45°20′ N and 84°45′ - 86°40 E) Prefecture (NX, Northern Xinjiang). Then three locations have Hami melon orchards with a planting area of not less than 3 ha were selected from each prefecture for sampling, and soil samples were collected in triplicates from each orchard (Fig. 1). In total, 54 rhizosphere soil samples were studied. The five-point sampling method was used for sample collection. Briefly, five Hami melons at maturity were randomly selected from each orchard to collect soil samples around their roots, at approximately 10 cm depth, using a shovel and sieved to remove plant residues and stones. The rhizosphere soil samples of five Hami melon plants were then mixed evenly and divided into three equal portions. Each sample was stored individually in sterile self-sealing bags and transported to the laboratory in an ice box (< 10 °C). After each soil sample was crushed and filtered using a 2 mm sieve, they were divided into two parts: one part was air dried and used for soil physicochemical analysis; the other part was stored in a − 80 °C refrigerator for DNA extraction.

The soil types in Kashgar, Aksu, Turpan and Hami Prefecture are clay loam, brown-gray clay loam, sandy loam and sandy clay loam, respectively. The soil types in Changji and Shihezi Prefecture are both loamy clay. Xinjiang has a variety of climate types, with a clear distinction between warm, cold and temperate from south to north, and dry and wet from east to west. Therefore, we divided all samples into Southern (SX), Eastern (EX) and Northern Xinjiang (NX) groups according to their geographical distribution for subsequent analysis. The climate information for each sampling site is shown in Table 5. The data of precipitation (PRCP) and temperature (TEMP) from NOAA - Climate Prediction Center (https://www.cpc.ncep.noaa.gov/), land surface temperature (LST) and relative humidity (RH) from NASA GES DISC MERRA2 - inst1_2d_asm_Nx (https://disc.gsfc.nasa.gov/), net solar radiation intensity (SWGNT) from NASA GES DISC MERRA2 - tavg1_2d_rad_Nx (https://disc.gsfc.nasa.gov/).

### DNA extraction and Illumina MiSeq

E.Z.N.A.® soil DNA Kit (Omega Biotek, USA) was used to extract total DNA from soil samples (0.5 g) following the manufacturer’s protocol. The final DNA concentration was detected using a NanoDrop 2000 UV-Vis spectrophotometer (Thermo Scientific, USA). The integrity of the DNA was assessed using 1% agarose gel electrophoresis. The yeast 26S rDNA was amplified with a pair of specific primers with barcode NL1F (forward primer) (5′-GCATATCAATAAGCGGAGGAAAAG-3′) and NL2R (reverse primer) (5′-CTTGTTCGCTATCGGTCTC-3′) [61]. The PCR reaction system (20 μL) contained 5× Fast*Pfu* Buffer (4 μL), 2.5 mM dNTPs (2 μL), primer (5 μM; 0.8 μL each), Fast*Pfu* Polymerase (0.4 μL), BSA (0.2 μL), and template DNA (10 ng). The PCR reaction was performed using a thermocycler PCR system as follows: 5 min at 98 °C (denaturation), 30 cycles at 98 °C for 30 s, 52 °C for 30 s, and 72 °C for 45 s, and finally, at 72 °C for 5 min (elongation). The PCR products were analyzed using 2% agarose gel electrophoresis, purified using the AxyPrep DNA Gel Extraction Kit (Axygen Biosciences, USA). The DNA fragments were quantified using QuantiFluor™-ST (Promega, USA) [62]. Equimolar amounts of purified DNA fragments were pooled after individual samples were tagged with indexes through an index PCR, and the Illumina MiSeq PE300 platform (Illumina, USA) was used to perform paired-end sequencing (2 × 300) following the protocol by Meiji Biomedical Technology Co. Ltd. (Shanghai, China).

### Sequence processing

Raw sequence files were demultiplexed and quality filtered by Trimmomatic and merged by FLASH based on the following criteria: (i) reads with an average quality score < 20 over a 50-bp sliding window were truncated; (ii) sequences with an overlap longer than 10 bp were merged based on their overlapping sequences; (iii) the maximum mismatch ratio allowed in the overlap region of a spliced sequence was 0.2, and non-conforming sequences were eliminated; (iv) the samples were differentiated according to the barcode and primers at the beginning and end of the sequence; the sequence orientation was adjusted, the number of mismatches allowed by the barcode was 0, and the maximum number of primer mismatches was 2 [63–65]. OTUs were clustered with a 97% similarity cutoff using UPARSE (version 7.1 http://drive5.com/uparse/), and chimeric sequences were identified and removed using the UCHIME software [66, 67]. The classification of each D1 domain of the LSU rRNA sequence was analyzed by the Ribosomal Database Project (RDP) Classifier algorithm (version 2.2 http://sourceforge.net/pro-jects/rdp-classifier/) [66]. The NCBI database (National Centre for Biotechnology Information, https://www.ncbi.nlm.nih.gov/public/) database using a confidence threshold of 0.7 [68]. The observed richness (Sobs), the ACE index the Chao1 estimator, the Shannon diversity (H) index and the Simpson index were calculated using the mothur (version v.1.30.2 https://mothur.org/wiki/chao/, https://mothur.org/wiki/ace/, https://mothur.org/wiki/shannon/, http://mothur.org/wiki/Simpson) index analysis with Operational Taxonomic Units (OTUs) at 0.97 level [69]. Next, we plotted the rarefaction curves to observe the community abundance of each sample and the sequencing data [62, 66].

### Determination of soil chemical properties

Here, we evaluated nine soil physicochemical factors (Table 4). The soil water suspension was shaken for 30 min, followed by measurement of pH using a glass electrode meter. A naturally dried soil sample was mixed with water at a ratio of 1:5 (M/V), and conductivity (CO) was determined using the electrode method. The organic matter (OM) was determined by titration with ferrous sulfate, using o-phenanthroline as the indicator, by adding a potassium dichromate-sulfuric acid solution to a test tube containing the soil samples. The available nitrogen (AN) and total nitrogen (TN) were determined by the Kjeldahl method. The available phosphorus (AP) in the soil was extracted with sodium bicarbonate and then determined using the molybdenum blue method. The available potassium (AK) in the soil was extracted with ammonium acetate and determined by flame photometry. Total phosphorus (TP) and total potassium (TK) were measured by acid solubilization [70, 71].

### Data analysis

SPSS Statistics v25.0 software (IBM, USA) was used to analyze the data of soil physicochemical properties and climatic factors. All values are presented as mean ± standard error (mean ± SE). Since the data were not normally distributed, Kruskal-Wallis test for independent samples was used to compare the physicochemical properties of the soil and climatic factors among different groups. Differences were taken statistically significant at *P* < 0.05. The dilution curve was drawn using the “vegan” and “ggplot2” packages in R (v4.0.2); Venn diagram using the “VennDiagram” package; community bar graph was plotted using “ggplot2” and “ggalluvial” packages in R (v4.0.2). Since the data of alpha diversity indices did not follow a normal distribution, the Kruskal-Wallis test was used to detect whether there were significant differences in alpha diversity indices among the groups. Analysis of the species that showed differences between groups based on genus level and phylum level was performed by Kruskal-Wallis rank-sum test, followed by plotting through the “ggplot2” package in R (v4.0.2). In this process the *P*-values are corrected for multiple testing by the false discovery rate (FDR) and further testing by Post-hoc testing after the Kruskal-Wallis H-test, with a further two-way comparison of the multiple groups, which is done by the stats package for R and the scipy package for Python. Principal co-ordinate analysis (PCoA) was done based on Bray-Curtis at OTU level to analyze similarities or differences in the community composition of samples using “vegan” and “ape” packages in R (v4.0.2). Tests for differences between groups in PCoA were analyzed using ANOSIM (analysis of similarities) by vegan package in R. Redundancy analysis (RDA) was used to evaluate the relationships between soil factors and yeast communities and between climatic factors and yeast communities respectively, based on sample soil physicochemical properties, local meteorological data and sample genus level data and calculated using the software Canoco for Windows 5 (Microcomputer Power, USA) [62, 65, 66]. Monte Carlo permutation test in Canoco was used to identify environmental factors that were significantly associated with yeast community structure.

## Supplementary Information

**Additional file 1: Fig. S1.** Box plot of Principal Coordinates analysis (PCoA) based on Bray-Curtis distance method at the OTU level. Red, blue and green represent samples from SX, EX, and NX, respectively. and the box plots in the figure represent the discrete distribution of different groups of samples on the PCoA1 axis.

**Additional file 2.**

## Data Availability

Sequence data of this project have been deposited in the Sequence Read Archive (SRA) of the National Center for Biotechnology Information (NCBI) under the accession number PRJNA725370 (https://www.ncbi.nlm.nih.gov/sra/?term=PRJNA725370).

## Abbreviations

SX: Southern Xinjiang
EX: Eastern Xinjiang
NX: Northern Xinjiang
OTU: Operational taxonomic units
CO: Conductivity
OM: Organic matter
TN: Total nitrogen
TP: Total phosphorus
TK: Total potassium
AN: Available nitrogen
AP: Available phosphorus
AK: Available potassium
PRCP: Average annual precipitation
TEMP: Average annual temperature
LST: Average annual land surface temperature
RH: Average annual relative humidity
SWGNT: Average annual net solar radiation intensity
PCoA: Principal coordinates analysis
RDA: Redundancy analysis
