# Supplementary figures and images for: Patterns of yeast diversity distribution and its drivers in rhizosphere soil of Hami melon orchards in different regions of Xinjiang
Source: BMC Microbiol. 2021 Jun 6;21:170. doi: 10.1186/s12866-021-02222-1 (PMC8180054; doi:10.1186/s12866-021-02222-1)

**R=0.6144, P=0.001**

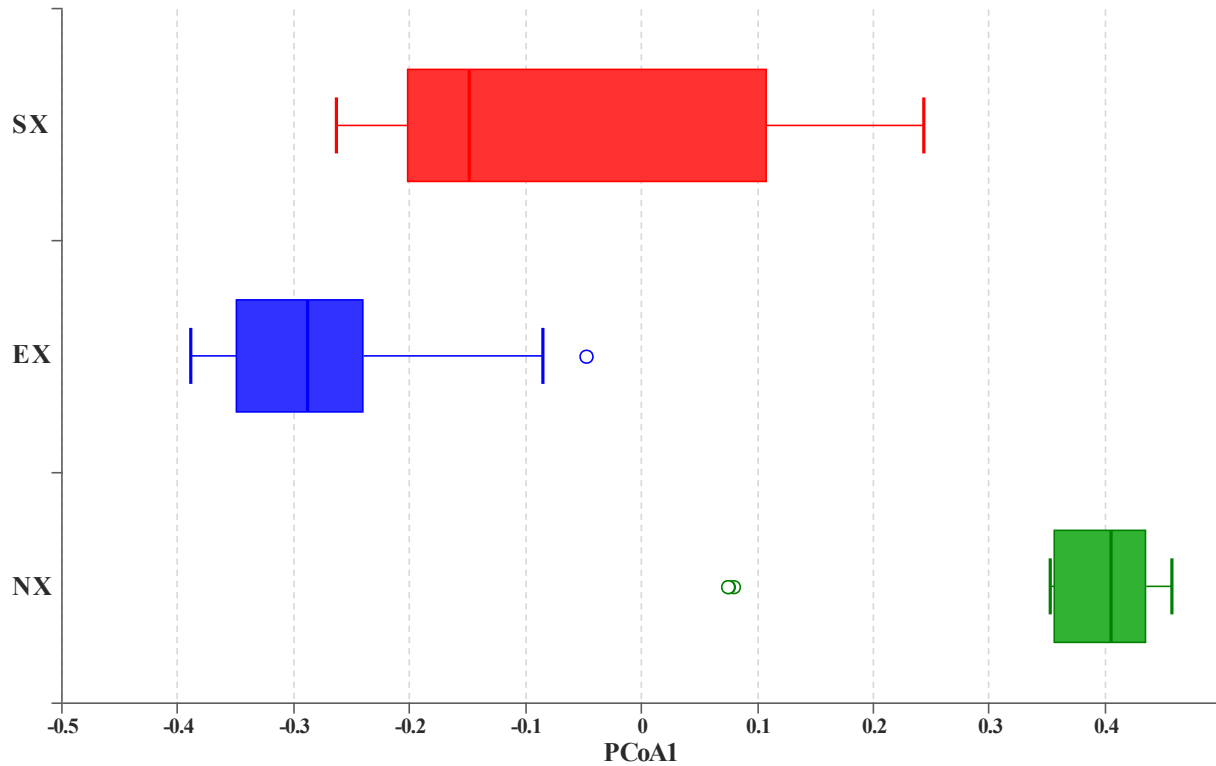

Supplement: Supplementary file 1 — Additional file 1: Fig. S1. Box plot of Principal Coordinates analysis (PCoA) based on Bray-Curtis distance method at the OTU level. Red, blue and green represent samples from SX, EX, and NX, respectively. and the box plots in the figure represent the discrete distribution of different groups of samples on the PCoA1 axis. [file 12866_2021_2222_MOESM1_ESM.pdf]
