# Supplementary material for: Patterns of yeast diversity distribution and its drivers in rhizosphere soil of Hami melon orchards in different regions of Xinjiang
Source: BMC Microbiol. 2021 Jun 6;21:170. doi: 10.1186/s12866-021-02222-1 (PMC8180054; doi:10.1186/s12866-021-02222-1)
Supplement: Supplementary file 2 — Additional file 2. [file 12866_2021_2222_MOESM2_ESM.docx]

**Table S1 Monte Carlo Permutation test for correlation between soil factors and yeast community**

| **Soil factors** | **F-ratio** | ***P*-value** |
| --- | --- | --- |
| pH | 6.04 | 0.059 |
| CO | 5.52 | 0.041* |
| OM | 2.13 | 0.136 |
| TN | 3.79 | 0.116 |
| TP | 6.02 | 0.001* |
| TK | 8.00 | 0.040* |
| AN | 2.54 | 0.369 |
| AP | 4.41 | 0.797 |
| AK | 4.73 | 0.107 |

Note: The abbreviations of soil factors are as in Table 4. Correlations are marked as ** for highly significant (P < 0.01) and * for significant (P < 0.05)

**Table S2 Monte Carlo Permutation test for correlation between** **climate factors and yeast community**

| **climate factors** | **F-ratio** | ***P*-value** |
| --- | --- | --- |
| PRCP | 15.79 | 0.003** |
| TEMP | 3.00 | 0.109 |
| LST | 3.06 | 0.387 |
| RH | 15.98 | 0.001** |
| SWGNT | 4.74 | 0.019* |

Note: The abbreviations of climate factors are as in Table 5. Correlations are marked as ** for highly significant (P < 0.01) and * for significant (P < 0.05)
